# University students’ overreliance on generative AI feedback in the learning process: the effects of perceived cognitive load, cognitive offloading, and metacognitive monitoring

**DOI:** 10.3389/fpsyg.2026.1998976

**Published:** 2026-10-28

**Authors:** Yuanyuan Guo, Peng Dong

**Affiliations:** 1 School of Law, Ningbo University, Ningbo, China; 2 Tsinghua University, Beijing, China

**Keywords:** cognitive load, cognitive offloading, cognitive regulation, educational psychology, metacognitive monitoring, digital society

## Abstract

Generative artificial intelligence (GAI) has introduced an important question in education, when AI provides immediate and reliable feedback, do students use it as a learning support tool or gradually rely on it as a substitute for their own cognitive efforts? Drawing on Cognitive Load Theory, Cognitive Offloading Theory, and Metacognitive Monitoring Theory, this study develops a theoretical model to explain the formation process of undergraduate students’ overreliance on GAI feedback. Specifically, this study examines the relationships among perceived cognitive load, cognitive offloading, and overreliance on GAI feedback, while further exploring how metacognitive monitoring levels shape this process. A questionnaire survey was conducted among 600 undergraduate students, and structural equation modeling was performed using AMOS 31.0. The results showed that perceived cognitive load significantly and positively predicted cognitive offloading, which further contributed to students’ overreliance on GAI feedback. Meanwhile, perceived cognitive load also directly predicted overreliance on GAI feedback. Bootstrap analysis further confirmed that cognitive offloading mediated the relationship between perceived cognitive load and overreliance on GAI feedback. In addition, this study established separate structural equation models for students with different levels of metacognitive monitoring and found distinct patterns in the formation of GAI feedback overreliance. Specifically, students with lower metacognitive monitoring were more likely to be influenced by cognitive pressure during learning, showing a more prominent direct relationship between perceived cognitive load and GAI feedback overreliance. This finding suggests that these students tend to rely more directly on GAI feedback when facing higher cognitive demands. In contrast, students with higher metacognitive monitoring demonstrated a more evident cognitive regulation process, in which cognitive offloading played a more important role in linking perceived cognitive load with GAI feedback overreliance. This indicates that these students may actively adjust their cognitive resource allocation when using GAI, although continuous cognitive offloading may still increase their dependence on AI-generated feedback.

## 1 Introduction

University learning requires students not only to acquire knowledge but also to regulate cognitive resources, evaluate information quality, and develop independent problem-solving abilities. With the rapid integration of generative artificial intelligence (GAI) into higher education, the way students obtain learning support has changed significantly. Unlike traditional digital learning tools that mainly provide information retrieval functions, GAI can generate explanations, feedback, examples, and suggestions based on students’ needs, offering continuous support similar to intelligent tutoring systems (Yan et al., 2024; Law, 2024). In tasks such as academic writing, knowledge understanding, problem solving, and learning feedback, GAI shows the potential to improve learning efficiency and support personalized learning (Iqbal et al., 2025; Evmenova et al., 2024). Therefore, the role of GAI in education has become an important topic in academic discussions.

On the one hand, appropriate use of generative AI tools, such as ChatGPT, can support knowledge exploration, improve learning efficiency, and promote the development of critical thinking (Guo and Lee, 2023; Suriano et al., 2025). By providing immediate explanations and continuous support, GAI helps students overcome limitations of classroom time and available resources and obtain more flexible learning assistance (Tlili et al., 2023; Ng et al., 2025). On the other hand, GAI may also change how students engage with feedback and process knowledge. Feedback serves as an important mechanism in learning because it helps students identify gaps in understanding, adjust learning strategies, and develop knowledge. However, feedback only improves learning when students understand, evaluate, and apply the provided information (Shute, 2008). In GAI-supported learning environments, automated feedback allows students to quickly obtain explanations and suggestions, but it may also reduce their opportunities to analyze, evaluate, and verify feedback actively. As a result, some students may accept AI-generated content without sufficient judgment (Yan et al., 2025; Bauer et al., 2025). When students rely excessively on AI-generated answers and feedback, they may reduce information searching, analytical thinking, and evaluation processes, which may weaken their monitoring of understanding and affect the development of critical thinking, independent judgment, and problem-solving abilities (Fan et al., 2025; Zhai et al., 2024).

These contrasting possibilities suggest that the influence of GAI on learning outcomes depends not only on the availability of AI tools but also on how students cognitively engage with, evaluate, and regulate AI-generated information. Existing studies suggest that technology dependence does not simply result from tool availability but is influenced by students’ cognitive demands, task pressure, trust in technology, and self-regulation abilities (Zhang et al., 2024; Mariñas et al., 2025). When students face difficult learning tasks, limited cognitive resources may encourage them to seek external support that reduces mental effort. Because GAI feedback can quickly provide structured responses and explanations, students may view it as an efficient cognitive resource (De Smedt et al., 2018; Butterfuss and Kendeou, 2018). Therefore, dependence on GAI feedback may not only reflect students’ preference for technology but also relate to how they manage cognitive resources during learning. Therefore, understanding students’ cognitive regulation processes is critical for explaining not only why some students develop overreliance on GAI feedback but also why the learning benefits of GenAI may vary across individuals and learning contexts.

Recent evidence suggests that the educational benefits of GenAI should be interpreted cautiously, as the current literature on AI-supported learning may be subject to publication bias, with positive effects being more likely to be reported than null or negative findings. Beyond the availability of AI tools, the learning value of GenAI depends on how students cognitively engage with, evaluate, and regulate AI-generated information. Although GenAI can provide immediate and personalized feedback, students need sufficient feedback literacy to interpret and apply such feedback effectively (Hawkins et al., 2025). Moreover, the contribution of GenAI to higher-order cognitive development appears to depend on its integration with appropriate pedagogical approaches rather than AI use alone (Li et al., 2026). Importantly, increased efficiency in completing learning tasks does not necessarily translate into improved learning outcomes, as students may complete tasks faster without achieving deeper cognitive processing (Molerov et al., 2026). Therefore, examining how students regulate their use of GAI feedback during learning is important for explaining why AI may support learning for some students but lead to overreliance for others.

One important context in which this issue becomes particularly relevant is university education, where students frequently encounter complex tasks requiring substantial cognitive resources (Sweller et al., 2019a,b; Risko and Gilbert, 2016). This cognitive regulation issue becomes particularly important in university education because students frequently encounter complex tasks, including academic writing, theoretical analysis, research design, and interdisciplinary problem solving. These tasks require students to process large amounts of information and engage in higher-level cognitive activities. Complex learning tasks can increase information-processing demands and influence students’ learning strategies and performance (De Smedt et al., 2018; Butterfuss and Kendeou, 2018). When students experience high cognitive demands, they may adopt strategies that reduce immediate mental effort, including using external tools to support or replace part of the cognitive work required for task completion (Sweller et al., 2019a,b; Risko and Gilbert, 2016). This tendency is also relevant in digital learning environments, where easy access to external information sources may reduce individuals’ reliance on internal cognitive resources (Sparrow et al., 2011). In GAI-supported learning contexts, a similar process may occur, as students may transfer parts of their understanding, judgment, and feedback evaluation processes to AI systems (Sparrow et al., 2011).

However, the extent to which such cognitive transfer leads to productive AI use or overreliance may depend on students’ ability to regulate their own learning processes. Students who can effectively monitor their understanding and evaluate external information may use GAI as a supportive resource, whereas insufficient regulation may increase dependence on AI-generated feedback. This highlights the importance of examining the cognitive mechanisms that shape students’ interaction with GAI. Despite increasing research interest in GAI-supported learning, several important issues remain unresolved. First, previous studies have mainly examined students’ attitudes toward AI, technology acceptance, or academic outcomes, while fewer studies have investigated the cognitive mechanisms that lead students to over-rely on AI feedback during learning activities (Klarin et al., 2024; Mariñas et al., 2025). Second, existing research on cognitive offloading has largely focused on traditional external tools, such as reminders, memory aids, and information systems. However, GAI represents a fundamentally different type of cognitive support because it can generate explanations, evaluate responses, and provide recommendations that directly influence learning decisions (Peng and Yeh, 2025; Seung and Basham, 2026). Third, limited attention has been given to the role of learner characteristics in shaping the relationship between cognitive demands, AI use, and dependence patterns (Yan et al., 2025; Bauer et al., 2025).

This study contributes to research on AI-supported learning by shifting attention from whether students use GAI to how they cognitively engage with AI-generated feedback. The present study focuses on university students and examines the relationships among perceived cognitive load, cognitive offloading, and overreliance on GAI feedback during learning. Specifically, this study investigates whether perceived cognitive load is associated with students’ tendency to rely on GAI feedback through cognitive offloading and further compares the structural relationships among these variables across different levels of metacognitive monitoring. By grouping students according to their metacognitive monitoring levels, this study aims to clarify whether students with different levels of metacognitive monitoring exhibit different patterns in managing cognitive resources when interacting with GAI.

## 2 Literature review

### 2.1 Theoretical foundation

This study adopts Cognitive Load Theory and Cognitive Offloading Theory as its theoretical foundations to explain university students’ overreliance on generative artificial intelligence (GAI) feedback. The integration of these two perspectives provides a more comprehensive explanation of students’ interactions with GAI during learning because neither theory alone can fully explain why students turn to AI feedback and how supportive AI use may gradually develop into excessive dependence. Cognitive Load Theory explains the cognitive demands that motivate students to seek external support, whereas Cognitive Offloading Theory explains the process through which students transfer cognitive activities to external tools. Together, these perspectives clarify the cognitive pathway from experiencing learning difficulties to relying on GAI feedback.

Cognitive Load Theory provides the initial explanation for why students may seek assistance from GAI during learning. The theory proposes that human working memory has limited capacity, and learning becomes more difficult when information processing demands exceed available cognitive resources (Sweller et al., 2019a,b). In university learning environments, students often encounter complex tasks that require simultaneous information processing, reasoning, evaluation, and knowledge integration, such as academic writing, theoretical analysis, and problem solving. These tasks may increase students’ perceived cognitive demands and influence how they allocate limited cognitive resources. When students experience excessive cognitive burden, they may search for strategies that reduce unnecessary mental effort and support task completion. However, Cognitive Load Theory alone cannot explain why students choose GAI rather than other forms of learning support, nor can it explain why reducing cognitive effort sometimes promotes learning while sometimes leads to dependence. The theory mainly focuses on the relationship between cognitive demands and learning efficiency, but it does not sufficiently explain how learners transfer cognitive activities to external resources. Therefore, another theoretical perspective is needed to understand the mechanism through which students use GAI feedback as an external cognitive support.

Cognitive Offloading Theory addresses this limitation by explaining how individuals reduce internal cognitive demands through external tools and environmental resources (Risko and Gilbert, 2016). Cognitive offloading represents a common human strategy for managing limited cognitive capacity. Individuals do not always complete cognitive tasks internally; instead, they strategically use external resources to store information, reduce memory demands, or improve task performance. Previous research has shown that cognitive offloading can be beneficial when external tools support learning activities and allow individuals to devote more resources to higher-level thinking (Gilbert et al., 2020; Grinschgl and Neubauer, 2022). The emergence of GAI, however, has expanded the meaning of cognitive offloading in educational contexts. Unlike traditional external tools that mainly assist memory, organization, or information retrieval, GAI can generate explanations, evaluate responses, provide suggestions, and produce solutions. Therefore, GAI may support not only peripheral cognitive activities but also core learning processes, including understanding, reasoning, and evaluation (Yan et al., 2024; Seung and Basham, 2026). This unique characteristic creates a possibility that students may move beyond appropriate cognitive support and gradually transfer essential cognitive responsibilities to AI systems.

Although Cognitive Offloading Theory explains how students transfer cognitive activities to GAI, it cannot independently explain why students decide to initiate such transfer. Cognitive offloading is not simply a result of tool availability; it often occurs when individuals perceive that internal cognitive effort is costly or insufficient for completing a task. Therefore, Cognitive Load Theory and Cognitive Offloading Theory address different but complementary aspects of GAI-supported learning. Cognitive Load Theory explains the psychological conditions that increase students’ motivation to reduce cognitive demands, while Cognitive Offloading Theory explains the behavioral process through which students use external tools to achieve this reduction. The combination of these two theories is particularly valuable for understanding overreliance on GAI feedback. If only Cognitive Load Theory is considered, students’ use of GAI may be interpreted merely as a reasonable response to increased task difficulty, without explaining why some students become excessively dependent on AI-generated feedback. If only Cognitive Offloading Theory is considered, the theory can describe students’ transfer of cognitive activities but cannot fully explain why cognitive demands trigger such behavior. By integrating both perspectives, this study views GAI feedback overreliance as a cognitive process in which perceived cognitive demands encourage students to reduce mental effort through external support, and this support may become problematic when students increasingly rely on AI to perform essential cognitive activities.

Therefore, this study integrates Cognitive Load Theory and Cognitive Offloading Theory to develop the *Cognitive Resource Management Framework for GAI Feedback Overreliance*, which explains how university students gradually shift from appropriate use of generative artificial intelligence (GAI) feedback to excessive dependence on such feedback. The framework argues that overreliance on GAI feedback does not result directly from the availability of AI tools. Instead, it emerges from the interaction among students’ cognitive demands, their strategies for managing cognitive resources, and their increasing reliance on external intelligent support during learning (Figure 1).

**Figure 1:**
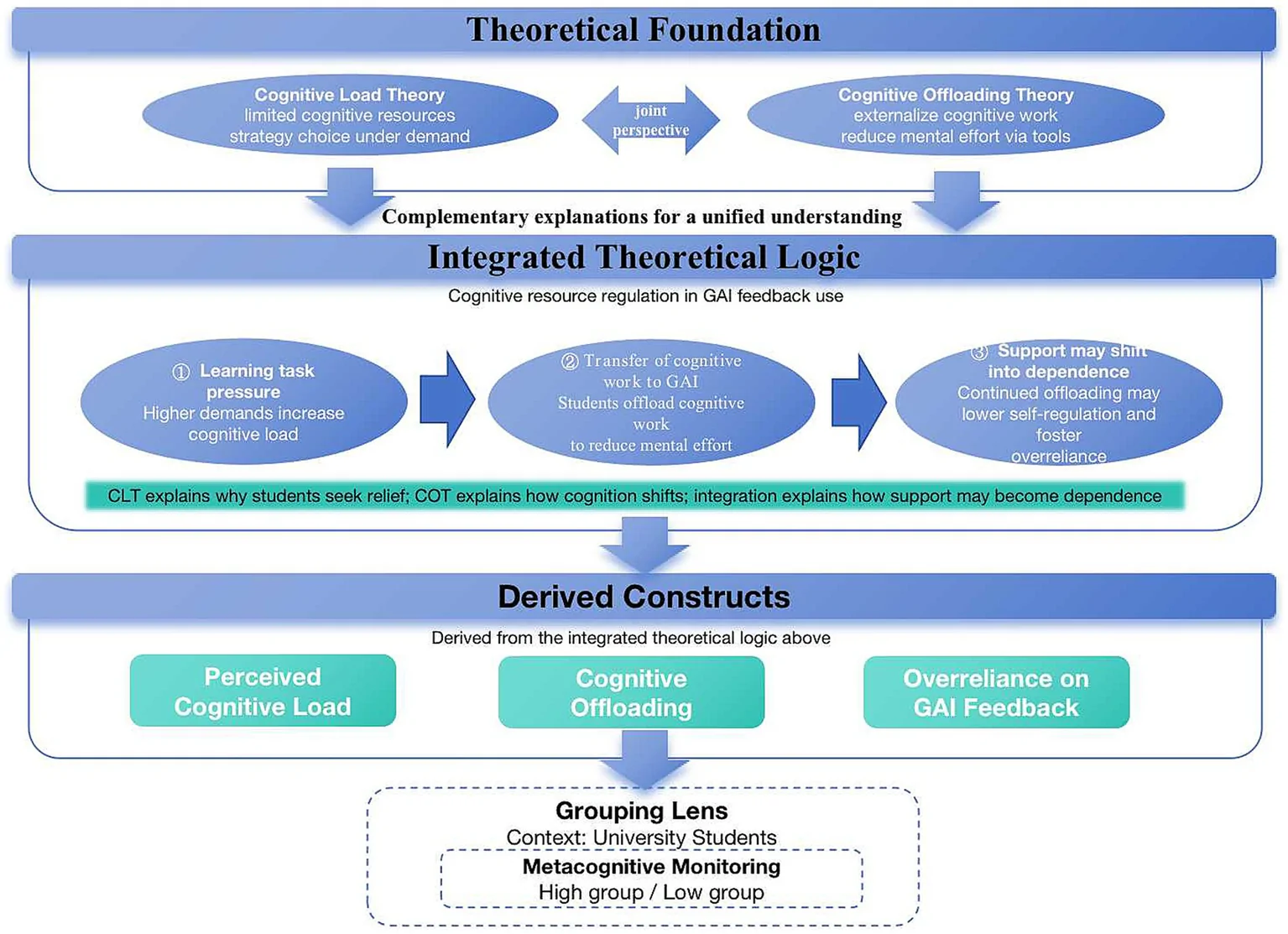
Cognitive resource management framework for GAI feedback overreliance.

Based on this framework, the formation of GAI feedback overreliance involves three connected cognitive processes, the emergence of cognitive demands, the redistribution of cognitive activities, and the increasing dependence on external cognitive support. First, when university students encounter complex learning tasks, higher cognitive demands may increase their need to reduce mental effort and seek external assistance. Therefore, students’ perceived cognitive pressure during learning represents an important starting point for explaining their subsequent behavioral choices. Second, when students attempt to manage such cognitive pressure, they may transfer part of their learning-related cognitive activities to GAI, forming cognitive offloading behaviors. Unlike traditional learning tools, GAI can generate explanations, feedback, and answers directly, which allows cognitive offloading to extend beyond simple assistance and involve essential learning processes that normally require students’ active engagement. Third, when students repeatedly rely on GAI to interpret feedback and make learning decisions, external cognitive support may gradually replace internal cognitive processing, resulting in overreliance on GAI feedback.

Following this cognitive process, this study does not regard GAI feedback overreliance as a simple outcome of technology use. Instead, it considers overreliance as the final expression of how students manage cognitive resources during learning. Accordingly, this study focuses first on students’ perceived cognitive demands, namely perceived cognitive load, to explain why students seek external cognitive support. It then examines cognitive offloading as the process through which students transfer part of their learning-related cognitive activities to GAI. Finally, it investigates whether this cognitive transfer develops into overreliance on GAI feedback, reflected in students’ reduced engagement in independent analysis, evaluation, and reflection.

However, this integrated framework mainly explains the common cognitive pathway through which students may develop dependence on GAI feedback. It cannot fully explain why students with similar cognitive demands and similar access to AI tools may show different levels of dependence. Students’ responses to GAI feedback also depend on whether they can monitor their understanding, evaluate external information, and adjust their learning strategies. Therefore, this study introduces metacognitive monitoring as a grouping variable to examine differences among university students with different levels of metacognitive monitoring. Students with stronger metacognitive monitoring may treat GAI feedback as supplementary learning information and engage in verification and reflection. In contrast, students with weaker metacognitive monitoring may accept AI feedback more directly and become more likely to rely excessively on AI-generated suggestions.

Therefore, the proposed framework explains how university students develop overreliance on GAI feedback through the process of increased cognitive demands, cognitive activity transfer, and insufficient self-monitoring. It suggests that using GAI does not inevitably lead to dependence. Instead, overreliance may occur when students experience greater cognitive pressure, increasingly transfer learning-related cognitive activities to AI, and fail to maintain effective monitoring of their own learning processes. This framework provides the basis for examining the relationships among perceived cognitive load, cognitive offloading, GAI feedback overreliance, and differences across levels of metacognitive monitoring.

### 2.2 The effect of perceived cognitive load on overreliance on GAI feedback

Perceived cognitive load may directly influence university students’ overreliance on GAI feedback because students’ perceptions of cognitive demands can affect how they respond to learning difficulties and allocate cognitive effort. When students experience greater cognitive pressure during learning tasks, they may find it more difficult to maintain sufficient attention, evaluation, and problem-solving effort. In such situations, GAI feedback provides an easily accessible source of explanations and solutions, which may reduce students’ willingness to complete learning-related judgments independently. Therefore, perceived cognitive load may increase students’ tendency to rely excessively on GAI feedback during learning.

First, higher perceived cognitive load may reduce students’ capacity to independently process and evaluate learning information. University learning requires students to integrate complex information, construct understanding, and make judgments across different academic tasks. When students perceive that task demands exceed their available cognitive resources, they may experience greater difficulty maintaining active cognitive engagement (De Smedt et al., 2018; Butterfuss and Kendeou, 2018). Under such conditions, students may prefer external support that can simplify information processing and reduce immediate mental demands. Because GAI can rapidly provide organized explanations, summaries, and feedback, students experiencing high cognitive load may be more likely to accept AI-generated information rather than invest additional effort in independent analysis.

Second, higher perceived cognitive load may increase students’ reliance on GAI feedback by changing their evaluation of the usefulness of external assistance. Cognitive resources are limited, and students need to decide how much effort they should invest in processing information during learning. Research on cognitive offloading suggests that individuals tend to use external tools when these tools can reduce cognitive demands and support task completion (Risko and Gilbert, 2016; Gilbert, 2024). Compared with traditional learning tools, GAI can provide direct responses, explanations, and recommendations, making it a particularly attractive option when students experience cognitive pressure (Yan et al., 2024; Law, 2024). As students increasingly perceive GAI feedback as an efficient way to overcome learning difficulties, they may gradually reduce their reliance on personal reasoning and increase dependence on AI-generated suggestions.

Third, perceived cognitive load may weaken students’ motivation to verify and critically examine GAI feedback. Effective learning requires students to actively interpret feedback, identify possible problems, and determine whether external information matches their own understanding (Shute, 2008). However, students who experience high cognitive demands may focus more on completing tasks than on carefully evaluating feedback quality. Since GAI can generate fluent and coherent responses, students may perceive AI feedback as sufficiently reliable and reduce further checking behaviors (Athaluri et al., 2023; Lee et al., 2023). Previous studies have suggested that excessive reliance on AI systems may occur when students accept AI outputs without adequate evaluation, which may weaken independent judgment and critical thinking during learning (Fan et al., 2025; Zhai et al., 2024).

Fourth, perceived cognitive load may make students more vulnerable to the convenience of GAI feedback. GAI differs from traditional information tools because it can participate in multiple stages of learning, including explanation, reasoning, and suggestion generation (Grinschgl and Neubauer, 2022; Seung and Basham, 2026). When students experience learning pressure, this high level of support may encourage them to view GAI not only as a learning assistant but also as a substitute for parts of their own cognitive work. Although appropriate use of GAI can support learning, excessive reliance may occur when students repeatedly use AI feedback to avoid cognitive effort and reduce independent consideration of learning problems (Gerlich, 2025; Yan et al., 2025).

Overall, perceived cognitive load may directly contribute to university students’ overreliance on GAI feedback. Higher cognitive demands may increase students’ need for immediate external support, reduce their willingness to independently evaluate information, and strengthen their dependence on AI-generated suggestions. Therefore, perceived cognitive load may represent an important cognitive condition that shapes how students interact with GAI feedback during learning. Based on this reasoning, the following hypothesis is proposed:

*H1*: Perceived Cognitive Load positively influences university students’ Overreliance on GAI Feedback.

### 2.3 The effect of cognitive offloading on overreliance on GAI feedback

Cognitive offloading provides an important perspective for understanding how students manage cognitive demands during GAI-supported learning. Research shows that individuals do not always rely on internal cognitive abilities to complete tasks. Instead, they actively use external tools to reduce cognitive demands and improve task performance (Risko and Gilbert, 2016). Appropriate cognitive offloading allows learners to save limited cognitive resources and devote more attention to complex analysis and creative activities (Gilbert et al., 2020; Grinschgl and Neubauer, 2022). For example, students may use digital tools to record information, organize learning materials, or obtain learning prompts, which can improve efficiency and support task completion. However, generative AI differs from traditional cognitive tools because it can directly produce explanations, summaries, reasoning processes, and answers. As a result, GAI may not only support learning activities but also replace cognitive processes that students originally needed to complete themselves (Yan et al., 2024; Seung and Basham, 2026).

Therefore, cognitive offloading in GAI-supported learning has both beneficial and problematic consequences. On the positive side, appropriate use of cognitive offloading can reduce unnecessary mental effort and support effective learning. For example, Iqbal et al. (2025) found that generative AI tool use could improve academic achievement through shared metacognition and cognitive offloading among preservice teachers. However, when students fail to evaluate AI-generated information actively, cognitive offloading may gradually become dependence on AI feedback, causing students to reduce their own cognitive involvement. Seung and Basham (2026) argued that cognitive offloading in the age of generative AI is not only related to efficiency but also concerns whether learners maintain active participation in the learning process. Similarly, Peng and Yeh (2025) showed that trust in external tools influences cognitive offloading decisions, and individuals who perceive tools as highly reliable are more likely to reduce their internal cognitive effort.

Cognitive offloading may represent an important psychological pathway through which university students develop overreliance on GAI feedback. Although external tools can support learning efficiency, students may gradually transfer essential cognitive activities, such as organizing information, understanding concepts, and evaluating solutions, to GAI when they frequently rely on AI assistance. Therefore, cognitive offloading may not only help students manage learning tasks but also increase the possibility that they accept GAI feedback without sufficient independent processing.

First, cognitive offloading may directly reduce students’ involvement in cognitive activities that are essential for effective learning. Cognitive offloading refers to the process through which individuals use external tools to reduce internal cognitive demands and complete tasks more efficiently (Risko and Gilbert, 2016). In traditional learning contexts, students may use external supports to manage information or reduce unnecessary mental effort. However, GAI differs from ordinary cognitive tools because it can generate explanations, summaries, suggestions, and solutions rather than simply store or present information (Grinschgl and Neubauer, 2022). When university students use GAI to complete parts of understanding, information processing, or problem solving, they may gradually reduce their own cognitive engagement with learning materials.

Second, cognitive offloading may increase students’ tendency to accept GAI feedback without careful evaluation. Effective learning requires students to examine information, compare new knowledge with existing understanding, and judge whether feedback fits the learning goal. However, when students transfer these evaluative activities to GAI, they may reduce their responsibility for checking and interpreting AI-generated information. Dunn and Risko (2016) suggested that cognitive offloading involves decisions about whether individuals should invest effort internally or rely on external support. In the context of GAI-supported learning, students who frequently choose external support may become less willing to perform independent evaluation of AI feedback. Peng and Yeh (2025) further indicated that trust in external tools influences cognitive offloading decisions, suggesting that students who perceive AI systems as reliable may more easily reduce their own cognitive effort.

Third, cognitive offloading may encourage students to treat GAI feedback as a substitute for personal judgment rather than as additional learning support. The educational value of GAI depends on whether students use AI outputs as resources for reflection or as direct answers that replace their own thinking processes (Yan et al., 2024). When students rely on GAI to generate explanations, identify key information, or provide problem-solving directions, they may obtain immediate task completion benefits but reduce opportunities for independent reasoning. Seung and Basham (2026) emphasized that cognitive offloading in the era of generative AI concerns not only efficiency but also whether learners maintain active cognitive participation. Similarly, Fan et al. (2025) argued that frequent use of generative AI may reduce students’ metacognitive engagement and encourage passive acceptance of AI-generated information.

Fourth, cognitive offloading may strengthen overreliance on GAI feedback because it changes students’ learning habits. Repeated reliance on external cognitive support can create a pattern in which students increasingly expect AI assistance whenever they encounter learning difficulties. Gilbert (2024) explained that individuals select cognitive offloading strategies according to perceived effort and expected benefits. Since GAI can provide rapid responses with relatively little effort from students, university students may gradually develop a preference for AI-supported decision making. This tendency becomes more problematic when students use GAI not only to reduce unnecessary cognitive demands but also to avoid necessary learning processes, such as analysis, reflection, and verification (Gerlich, 2025; Zhai et al., 2024).

Moreover, previous studies on AI-supported learning indicate that cognitive offloading has a dual influence on students’ learning behaviors. Appropriate use of GAI can help students organize information and manage complex tasks, which may support learning efficiency (Iqbal et al., 2025). However, excessive cognitive outsourcing may weaken students’ independent thinking and increase dependence on AI-generated suggestions (Yan et al., 2025). Therefore, the key issue is not whether students use GAI, but whether they transfer essential cognitive responsibilities to AI during learning activities. When cognitive offloading exceeds a reasonable level, students may become increasingly dependent on GAI feedback and reduce their own evaluation and judgment processes. Based on this reasoning, cognitive offloading may serve as a direct psychological factor influencing university students’ overreliance on GAI feedback. Students who frequently transfer learning-related cognitive activities to GAI may become more likely to accept AI feedback without sufficient verification, depend on AI suggestions when making learning decisions, and reduce independent thinking during problem solving. Therefore, this study proposes the following hypothesis:

*H2*: Cognitive offloading positively influences university students’ overreliance on GAI feedback.

### 2.4 The effect of perceived cognitive load on cognitive offloading

Perceived cognitive load may influence university students’ cognitive offloading during GAI-supported learning. When students encounter complex learning tasks, they need to allocate limited cognitive resources to information processing, comprehension, and problem solving. If students perceive that task demands exceed their available cognitive capacity, they may seek external support to reduce immediate mental effort. Therefore, perceived cognitive load may increase students’ tendency to transfer part of their cognitive activities to external systems such as GAI.

First, perceived cognitive load may encourage students to use cognitive offloading as a strategy for managing limited cognitive resources. University students often complete tasks that require extensive information processing, such as academic writing, theoretical analysis, and complex problem solving. These tasks require students to integrate multiple sources of information and maintain continuous cognitive control. Previous research shows that demanding learning activities can increase cognitive challenges and influence students’ strategy choices during learning (De Smedt et al., 2018; Butterfuss and Kendeou, 2018). When students experience greater cognitive pressure, they may prefer strategies that reduce unnecessary mental effort. Cognitive offloading provides such support by allowing individuals to use external resources to decrease internal cognitive demands (Risko and Gilbert, 2016).

Second, perceived cognitive load may affect students’ evaluation of the usefulness of external cognitive support. Cognitive offloading does not occur randomly; individuals decide whether to rely on external tools according to task requirements, expected benefits, and cognitive costs (Gilbert, 2024). When university students perceive learning tasks as difficult, external tools become more attractive because they can help organize information, reduce memory demands, and provide guidance for completing tasks. In GAI-supported environments, students may use AI to arrange learning materials, explain difficult concepts, and generate possible solutions. Grinschgl and Neubauer (2022) suggested that modern technologies can function as cognitive partners that support human thinking. Thus, higher perceived cognitive load may increase students’ willingness to involve GAI in cognitive activities.

Third, perceived cognitive load may influence the depth and range of students’ cognitive offloading. Moderate use of external support can help students manage complex tasks effectively, but high cognitive pressure may lead students to transfer more important cognitive activities to external systems. Dunn and Risko (2016) explained that individuals make cognitive offloading decisions by balancing internal effort and external assistance. When students perceive that maintaining independent cognitive processing requires excessive effort, they may increasingly rely on external tools to complete understanding, information organization, and problem solving. In this process, perceived cognitive load may shift cognitive offloading from a supportive strategy toward a broader transfer of learning-related activities.

Fourth, GAI may strengthen the relationship between perceived cognitive load and cognitive offloading because it provides more comprehensive cognitive support than traditional tools. Traditional learning tools usually assist specific tasks, whereas GAI can generate explanations, summaries, and problem-solving suggestions according to students’ needs (Yan et al., 2024). This feature allows students to transfer not only simple information management tasks but also higher-level cognitive activities to AI systems. Seung and Basham (2026) noted that cognitive offloading in the era of generative AI concerns how learners distribute cognitive responsibilities between themselves and intelligent systems. Therefore, when students experience high cognitive load, GAI may become an especially appealing option for reducing mental demands.

Furthermore, students’ trust in external tools may strengthen their tendency to engage in cognitive offloading. Individuals are more likely to rely on external support when they believe that the tool can provide accurate and useful assistance. Weis and Wiese (2019) found that perceived reliability influences cognitive offloading decisions, and Peng and Yeh (2025) further showed that trust toward tools affects individuals’ willingness to reduce internal cognitive effort. In university learning contexts, students who experience high cognitive load and perceive GAI as reliable may show stronger intentions to transfer cognitive tasks to AI. However, cognitive offloading does not always represent ineffective learning behavior. Appropriate cognitive offloading can help students manage complex tasks and reserve cognitive resources for deeper learning activities. For example, Iqbal et al. (2025) found that generative AI use combined with cognitive offloading could support academic achievement among preservice teachers. The influence of perceived cognitive load on cognitive offloading therefore depends on how students use external support. When students use GAI to reduce unnecessary cognitive demands, cognitive offloading may support learning efficiency. When students use GAI mainly to avoid necessary thinking processes, cognitive offloading may reduce active cognitive engagement.

In summary, perceived cognitive load may serve as an important antecedent of cognitive offloading among university students. When students experience greater information-processing pressure and mental effort requirements, they may become more likely to rely on GAI to manage learning demands and transfer part of their cognitive activities to external systems. Based on this reasoning, this study proposes the following hypothesis:

*H3*: Perceived cognitive load positively influences university students’ cognitive offloading.

### 2.5 The mediating role of cognitive offloading between perceived cognitive load and overreliance on GAI feedback

Although perceived cognitive load may influence students’ overreliance on GAI feedback, this relationship may not occur directly. Cognitive offloading may explain how cognitive pressure is transformed into dependence on AI-generated feedback. In GAI-supported learning environments, students’ responses to cognitive demands depend not only on the level of difficulty they perceive but also on how they redistribute cognitive activities between themselves and external systems. Therefore, cognitive offloading may serve as a psychological pathway connecting perceived cognitive load with overreliance on GAI feedback. When students experience high cognitive demands, they may attempt to reduce cognitive burden by transferring part of learning-related activities to GAI. This process represents a change in the distribution of cognitive responsibility between students and external systems. Unlike traditional tools that mainly support information storage or retrieval, GAI can participate in understanding, explanation, summarization, and problem-solving processes (Grinschgl and Neubauer, 2022; Yan et al., 2024). As students increasingly rely on GAI to manage these cognitive activities, they may gradually reduce their involvement in evaluating and processing information independently.

The transition from cognitive support to cognitive dependence may explain why cognitive offloading contributes to overreliance on GAI feedback. Cognitive offloading itself does not necessarily lead to negative learning outcomes because external support can help students manage complex tasks effectively. However, when students transfer essential cognitive activities to GAI, they may reduce opportunities to construct knowledge, monitor understanding, and evaluate feedback quality. Risko and Gilbert (2016) argued that cognitive offloading reflects a balance between internal cognitive effort and external assistance, while Gilbert (2024) further suggested that individuals decide whether to rely on external resources according to perceived cognitive costs and expected benefits. In GAI-supported learning contexts, repeated cognitive outsourcing may gradually shape students’ dependence on AI feedback.

Furthermore, the characteristics of GAI may strengthen the mediating role of cognitive offloading. Because GAI can generate fluent explanations and seemingly complete solutions, students may perceive AI feedback as sufficient for addressing learning problems. When students have already transferred part of their cognitive work to GAI, they may become more likely to accept AI-generated suggestions without further verification. This process may weaken independent judgment and increase reliance on external feedback sources (Dunn and Risko, 2016; Fan et al., 2025). Seung and Basham (2026) further emphasized that cognitive offloading in the era of generative AI concerns whether learners maintain active participation in cognitive activities while using intelligent systems. Therefore, cognitive offloading may represent the mechanism through which perceived cognitive load develops into overreliance on GAI feedback. Higher perceived cognitive load may encourage students to transfer cognitive activities to GAI, and this increased cognitive offloading may subsequently strengthen their dependence on AI feedback during learning. In this process, cognitive offloading explains how students’ attempts to manage cognitive demands may unintentionally shift toward reduced independent evaluation and excessive reliance on GAI-generated information. Based on this reasoning, this study proposes the following hypothesis:

*H4*: Cognitive offloading mediates the relationship between perceived cognitive load and university students’ overreliance on GAI feedback.

The theoretical model of this study is presented in Figure 2.

**Figure 2:**
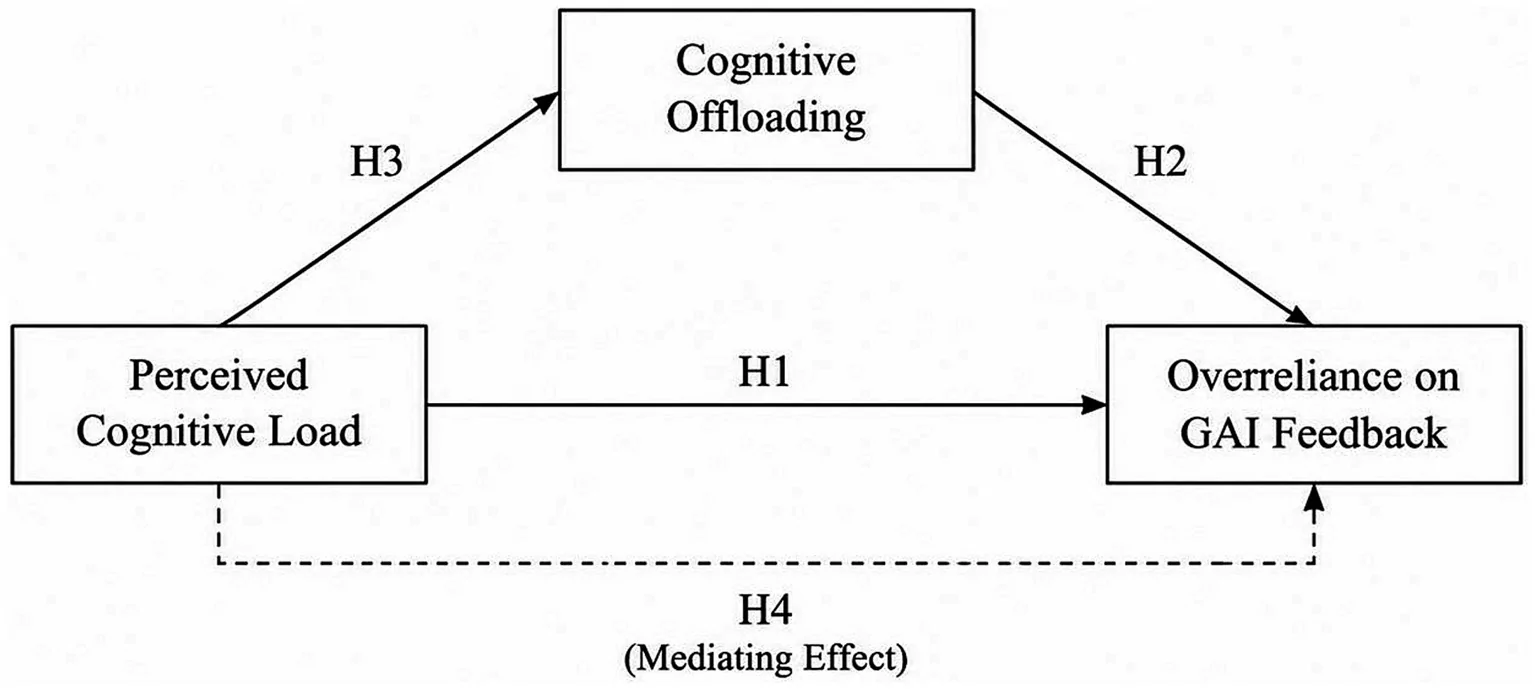
Theoretical model.

### 2.6 Grouping role of metacognitive monitoring

Pervious findings suggest that students’ perceived cognitive load may serve as an important starting point for understanding GAI feedback overreliance, while cognitive offloading may explain the process through which this relationship develops. However, students do not respond to cognitive pressure in the same way. Some students may use GAI as a supportive learning resource, whereas others may gradually rely on AI to complete essential cognitive activities. This difference may depend on students’ ability to monitor and regulate their own learning processes. Metacognitive monitoring enables learners to evaluate their understanding, judge the quality of external information, and adjust learning strategies accordingly (Zimmerman, 2002). In GAI-supported environments, students with stronger metacognitive monitoring abilities may treat AI feedback as supplementary information and engage in comparison, verification, and reflection. In contrast, students with weaker metacognitive monitoring may accept AI feedback without sufficient evaluation, increasing the possibility of overreliance (Dunn and Risko, 2016; Fan et al., 2025).

Although previous studies have examined the influence of generative AI on learning behaviors and cognitive processes, the psychological mechanisms that lead university students to over-rely on GAI feedback remain unclear. Existing research has mainly focused on students’ acceptance of AI tools, frequency of use, or learning outcomes, while less attention has been given to how students process AI feedback during actual learning activities and why they shift from using AI as learning support to depending on AI for cognitive work (Klarin et al., 2024; Mariñas et al., 2025). This issue is important because successful learning depends not only on obtaining correct information but also on students’ active involvement in understanding, evaluating, and integrating knowledge (Yan et al., 2025). Research on AI-supported learning also reveals a complex pattern of student behaviors. On one hand, GAI can reduce learning barriers, provide timely feedback, and support knowledge construction. For example, in writing education, generative AI can help students identify language problems, improve text organization, and develop better expressions (Evmenova et al., 2024; Goldman et al., 2025). In mathematics and problem-solving contexts, AI-generated feedback can provide additional explanations and support students’ understanding of difficult concepts (Cosentino et al., 2025). On the other hand, the high accessibility of AI feedback may reduce students’ active processing of information. When students focus mainly on completing tasks rather than understanding the reasoning behind feedback, AI may shift from a learning assistant to an external substitute for thinking (León-Domínguez, 2024; Fan et al., 2025).

This concern is especially relevant for university students because higher education requires learners to handle open-ended, uncertain, and cognitively demanding tasks. Activities such as academic writing, case analysis, research design, and complex problem solving require not only correct answers but also analysis, evaluation, and reflection (Butterfuss and Kendeou, 2018; Alkhatib, 2019). However, GAI can quickly generate responses that appear complete and logically organized, which may reduce students’ motivation to examine the accuracy, suitability, and limitations of AI feedback (Athaluri et al., 2023; Lee et al., 2023). When students regard AI feedback as an authoritative answer rather than learning material that requires further evaluation, overreliance may occur. To better understand this process, it is necessary to examine how students manage cognitive resources during learning. Perceived cognitive load may influence students’ decisions to seek external support because students who experience excessive mental demands may prefer strategies that reduce immediate cognitive effort (De Smedt et al., 2018). Since GAI can provide direct explanations, summaries, and feedback, it offers an attractive way to reduce cognitive demands. However, if students reduce cognitive effort mainly by transferring essential learning activities to AI, they may experience lower cognitive engagement and weaker knowledge construction (Risko and Gilbert, 2016; Gerlich, 2025).

Previous studies indicate that cognitive offloading itself is not a negative behavior but an adaptive strategy that helps individuals manage complex tasks. People decide whether to transfer cognitive activities to external tools based on task goals, expected benefits, and perceived tool reliability (Gilbert, 2024; Weis and Wiese, 2019). For example, using calendars to manage study schedules, search engines to obtain information, or software to perform calculations represents reasonable cognitive offloading. However, compared with traditional tools, GAI possesses stronger abilities in information generation and reasoning. Therefore, GAI-related cognitive offloading may involve more central learning activities, including understanding, explanation, evaluation, and decision making (Grinschgl and Neubauer, 2022; Seung and Basham, 2026). However, cognitive offloading does not inevitably result in overreliance on GAI feedback. The influence of cognitive offloading depends largely on whether students can regulate their own learning activities and evaluate external information effectively. Metacognitive monitoring plays a central role in this process because it allows learners to judge their current understanding, identify knowledge gaps, and decide whether additional cognitive effort is necessary (Zimmerman, 2002). In traditional learning environments, effective learners continuously monitor comprehension and adjust strategies according to task requirements. In AI-supported learning environments, these abilities become even more important because students must determine when AI assistance is beneficial and when independent thinking is required.

Metacognitive monitoring may therefore explain why students with similar levels of cognitive load and access to GAI feedback may develop different patterns of AI use. Students with strong metacognitive monitoring skills are more likely to question AI-generated responses, compare them with their own knowledge, and identify possible errors or limitations. Such students may use GAI as a cognitive partner that supports deeper learning rather than as a replacement for personal thinking (Shin, 2021; Buçinca et al., 2021). In contrast, students with insufficient metacognitive monitoring may accept AI feedback without careful evaluation because they perceive the generated information as accurate and reliable. This tendency may increase their dependence on AI-generated suggestions and reduce their engagement in independent reasoning (Fan et al., 2025). Recent studies have emphasized that the educational challenge of GAI is not whether students should use AI tools, but whether they can develop appropriate reliance on these technologies. Appropriate reliance requires learners to recognize the strengths and limitations of AI, determine when external support is needed, and maintain responsibility for evaluating information quality (Li and Little, 2023). This perspective shifts attention from simple AI adoption to the cognitive processes that shape how students interact with AI during learning. Understanding individual differences in metacognitive monitoring is therefore essential for explaining why some students benefit from GAI feedback while others become overly dependent on it.

Based on the above discussion, this study considers metacognitive monitoring as an important individual difference that may shape students’ interaction with GAI feedback. However, the present study does not aim to examine the moderating effect of metacognitive monitoring through an interaction model. Instead, metacognitive monitoring is used as a grouping lens to compare whether the proposed relationships among perceived cognitive load, cognitive offloading, and overreliance on GAI feedback are consistent across students with different levels of metacognitive monitoring. Specifically, students are divided into high and low metacognitive monitoring groups based on their responses to the metacognitive monitoring measure. By comparing these two groups, this study aims to explore whether students with different levels of self-monitoring ability show similar or different patterns in managing cognitive resources and using GAI feedback during learning.

## 3 Method

### 3.1 Data source

This study collected data from undergraduate students at Ningbo University, located in Jiangbei District, Ningbo, Zhejiang Province, China. The university was selected as the research setting because it provides a diverse undergraduate learning environment with students from multiple academic disciplines, allowing the study to examine students’ cognitive experiences across different learning tasks and academic contexts. As a comprehensive university, Ningbo University includes students who frequently engage in activities such as academic writing, information search, knowledge acquisition, and problem solving, all of which increasingly involve the use of generative artificial intelligence (GAI) tools. Therefore, the university context provides a suitable setting for investigating how undergraduate students perceive and use GAI feedback in learning processes.

The study adopted an online questionnaire survey method and collected data through the Credamo research platform from August 1 to August 25, 2026. Credamo was selected because it provides a standardized procedure for questionnaire distribution, participant screening, and response management, which helps improve the consistency and reliability of online data collection. Before data collection, the study established specific eligibility criteria to ensure that participants had sufficient experience with GAI-supported learning activities. Participants needed to meet two conditions: they had to be undergraduate students at Ningbo University, and they had to have previous experience using GAI tools for academic learning purposes.

To ensure that respondents could provide experience-based evaluations rather than general opinions about artificial intelligence, the questionnaire included a screening question regarding previous GAI use. Participants reported whether they had used commonly available GAI applications, including Doubao, DeepSeek, and ChatGPT. These platforms provide functions related to learning support, such as knowledge explanation, information summarization, writing assistance, and problem-solving guidance. Only students who reported actual experience using at least one GAI platform were retained for subsequent analysis.

The research team initially distributed 760 questionnaires through the Credamo platform. After completing data collection, the study conducted a multi-step data screening procedure to ensure data quality. First, questionnaires from participants who did not satisfy the sampling criteria were removed. Second, incomplete responses were excluded. Third, responses with abnormal completion patterns, including extremely short response times and identical answers across all measurement items, were removed to reduce the influence of careless responding. After the screening process, 600 valid questionnaires remained, producing a valid response rate of 78.95%.

The final dataset consisted of 600 undergraduate students with direct experience using GAI in learning contexts. These participants provided empirical information about how students experience cognitive demands, transfer cognitive activities to GAI systems, and develop different levels of reliance on GAI feedback. Although the sample came from a single university, the clear inclusion criteria, experience-based participant selection, and strict quality-control procedures ensured that the data were appropriate for examining the cognitive mechanisms associated with university students’ overreliance on GAI feedback.

### 3.2 Variable definitions and measurement items

This study included four variables to examine the cognitive process through which university students develop overreliance on GAI feedback during learning activities. Although previous studies have examined cognitive load, cognitive offloading, AI dependence, and metacognitive regulation, existing measurement scales were mainly developed for traditional learning environments or general technology use contexts. These scales do not directly capture how university students experience cognitive demands, transfer cognitive activities, and evaluate GAI-generated feedback in current AI-supported learning environments. Therefore, this study developed measurement items based on previous theoretical and empirical studies while adapting the wording to the specific context of university students’ interactions with GAI during learning.

Perceived Cognitive Load (PCL) was developed to assess students’ subjective perception of cognitive demands during learning tasks. Previous research on cognitive load emphasizes that students’ learning experiences depend not only on the objective complexity of learning materials but also on their perceived information-processing demands and required mental effort (Sweller et al., 2019a,b). Accordingly, this study measured whether students experience difficulty managing information, require substantial mental effort, and perceive complex relationships among learning contents.

Cognitive Offloading (CO) was developed to measure the extent to which students transfer cognitive activities from themselves to GAI systems during learning. Cognitive offloading research suggests that individuals often use external tools to reduce cognitive demands and improve task performance (Risko and Gilbert, 2016). However, unlike traditional tools that mainly store or retrieve information, GAI can generate explanations, organize knowledge, summarize information, and provide problem-solving suggestions. Therefore, this study focused on four forms of GAI-related cognitive offloading, including information organization, understanding, information extraction, and problem solving.

Overreliance on GAI Feedback (OGF) was developed to capture students’ excessive dependence on AI-generated feedback during learning. Previous research indicates that excessive reliance on AI systems may reduce independent evaluation, critical judgment, and active cognitive engagement (Zhai et al., 2024). Therefore, this study defined overreliance on GAI feedback as students’ tendency to accept, follow, or evaluate learning outcomes mainly according to GAI feedback rather than their own analysis and verification.

Metacognitive Monitoring (MM) was included as a grouping variable rather than a predictor or moderator. This variable reflects students’ ability to monitor their own understanding and evaluate the appropriateness of GAI feedback before acceptance. Research on self-regulated learning emphasizes that metacognitive monitoring helps students judge their comprehension, identify knowledge gaps, and adjust learning strategies (Zimmerman, 2002). In this study, MM was used to classify students into high and low metacognitive monitoring groups to examine whether students with different monitoring abilities show different patterns of GAI feedback use.

All measurement items were rated on a five-point Likert scale ranging from 1 (strongly disagree) to 5 (strongly agree). The measurement items were developed according to the conceptual boundaries of each variable, and each item focused on a distinct aspect of the construct to avoid overlap among variables. Table 1 presents the operational definitions, measurement items, measurement focus, and reference basis of the four variables.

**Table 1:** Measurement items.

Variable	Role in model	Operational definition	Item code	Measurement item	Measurement focus
Perceived Cognitive Load (PCL)	Independent Variable	Students’ perceived level of cognitive demands, mental effort, and information-processing pressure when completing learning tasks.	PCL1	I feel that some learning tasks require me to process more information than I can easily manage.	Information-processing pressure
			PCL2	I often need to make considerable mental effort to understand complex learning materials.	Mental effort requirement
			PCL3	I find that learning tasks often involve complex relationships among ideas that require substantial mental effort to understand.	Content complexity and cognitive burden
Cognitive Offloading (CO)	Mediating Variable	Students’ tendency to transfer part of their cognitive activities that would normally require their own mental effort to GAI during learning.	CO1	I use GAI to organize learning materials that would otherwise require my own effort to arrange.	Information organization offloading
			CO2	I use GAI to generate explanations that help me complete parts of the understanding process.	Understanding-process offloading
			CO3	I use GAI to summarize key information that I would otherwise need to identify and extract by myself.	Information extraction offloading
			CO4	I use GAI to provide initial ideas or directions when I need to solve learning problems.	Problem-solving offloading
Overreliance on GAI Feedback (OGF)	Dependent Variable	Students’ excessive dependence on GAI feedback during learning, reflected in reduced independent evaluation, verification, and judgment of AI-generated feedback.	OGF1	I usually accept GAI feedback without carefully checking whether it is correct.	Lack of feedback verification
			OGF2	I find it difficult to make learning decisions without relying on GAI feedback.	Decision dependence
			OGF3	I tend to follow GAI suggestions even when I have not independently considered other possible solutions.	Lack of independent thinking
			OGF4	GAI feedback has become my primary basis for judging whether my learning work is acceptable.	External evaluation dependence
Metacognitive Monitoring (MM)	Grouping Variable	Students’ ability to monitor their own understanding and evaluate the appropriateness of GAI feedback before accepting it. This variable is used to divide students into high and low metacognitive monitoring groups.	MM1	Before accepting GAI feedback, I usually evaluate whether it matches my own understanding.	Feedback evaluation monitoring

### 3.3 Sample information

Table 2 presents the demographic characteristics and GAI usage patterns of the 600 undergraduate students. Among the participants, 286 students were male (47.67%) and 314 students were female (52.33%). The sample included students from all four academic years, with 150 freshmen (25.00%), 153 sophomores (25.50%), 148 juniors (24.67%), and 149 seniors (24.83%). Regarding GAI use frequency, 48 students (8.00%) reported rarely using GAI tools, 112 students (18.67%) used them occasionally, 156 students (26.00%) used them sometimes, 178 students (29.67%) used them frequently, and 106 students (17.66%) used them very frequently. For the metacognitive monitoring grouping variable, 309 students (51.50%) were classified into the high metacognitive monitoring group, while 291 students (48.50%) were classified into the low metacognitive monitoring group.

**Table 2:** Descriptive statistical analysis.

Item	Option	Frequency	Percent (%)
Gender	Male	286	47.67
	Female	314	52.33
Academic Year	Freshman	150	25.00
	Sophomore	153	25.50
	Junior	148	24.67
	Senior	149	24.83
GAI Use Frequency	Rarely	48	8.00
	Occasionally	112	18.67
	Sometimes	156	26.00
	Frequently	178	29.67
	Very frequently	106	17.66
Group	High Metacognitive Monitoring Group	309	51.50
	Low Metacognitive Monitoring Group	291	48.50
Total		600	100.00

### 3.4 Item analysis

Before conducting reliability and validity analyses, this study performed item analysis to examine the discriminating ability of each measurement item. The critical ratio method was applied by dividing participants into high-score and low-score groups based on their total scores for each construct, and independent-samples t-tests were conducted to examine group differences. As shown in Table 3, all items of perceived cognitive load (PCL1–PCL3), cognitive offloading (CO1–CO4), and overreliance on GAI feedback (OGF1–OGF4) showed significant differences between the high-score and low-score groups (*p* < 0.001). The critical ratios ranged from 18.266 to 22.640 for cognitive offloading items, 18.724 to 20.831 for perceived cognitive load items, and 19.920 to 21.272 for overreliance on GAI feedback items. These results indicate that all measurement items could effectively distinguish participants with different levels of the corresponding constructs. Therefore, all items demonstrated satisfactory discriminating ability and were retained for subsequent reliability and validity analyses.

**Table 3:** Item analysis.

Construct	Item	Low-score group (mean ± S. D.)	High-score group (mean ± S. D.)	*t*-value	*p*-value
Perceived Cognitive Load (PCL)	PCL1	3.14 ± 0.83	4.50 ± 0.50	20.831	<0.001
	PCL2	3.20 ± 0.82	4.47 ± 0.50	19.587	<0.001
	PCL3	3.18 ± 0.86	4.44 ± 0.51	18.724	<0.001
Cognitive Offloading (CO)	CO1	2.73 ± 0.87	4.34 ± 0.55	22.640	<0.001
	CO2	3.23 ± 0.77	4.40 ± 0.52	18.266	<0.001
	CO3	2.88 ± 0.83	4.35 ± 0.52	21.861	<0.001
	CO4	3.14 ± 0.91	4.47 ± 0.50	18.670	<0.001
Overreliance on GAI Feedback (OGF)	OGF1	3.11 ± 0.82	4.60 ± 0.49	21.272	<0.001
	OGF2	2.95 ± 0.78	4.31 ± 0.54	20.638	<0.001
	OGF3	2.94 ± 0.84	4.36 ± 0.48	19.920	<0.001
	OGF4	2.91 ± 0.86	4.42 ± 0.55	20.299	<0.001

### 3.5 Reliability and validity testing

This study conducted reliability analysis to examine the internal consistency of the measurement scales. As shown in Table 4, the Cronbach’s α coefficients of perceived cognitive load (PCL), cognitive offloading (CO), and overreliance on GAI feedback (OGF) were 0.772, 0.803, and 0.805, respectively. All values exceeded the commonly accepted threshold of 0.70, indicating satisfactory internal consistency among the measurement items. Furthermore, the corrected item-total correlation (CITC) values ranged from 0.574 to 0.653, which indicates that all items showed adequate correlations with their corresponding constructs. The Cronbach’s α values after deleting any item did not exceed the overall reliability coefficients, suggesting that removing any item would not improve the reliability of the scales. Therefore, all measurement items demonstrated acceptable reliability and were retained for subsequent validity analysis and hypothesis testing.

**Table 4:** Reliability analysis results.

Construct	Item	Corrected item-total correlation (CITC)	Cronbach’s *α* if item deleted	Cronbach’s *α*
Perceived Cognitive Load (PCL)	PCL1	0.623	0.674	0.772
	PCL2	0.622	0.675	
	PCL3	0.574	0.728	
Cognitive Offloading (CO)	CO1	0.634	0.747	0.803
	CO2	0.624	0.754	
	CO3	0.628	0.748	
	CO4	0.593	0.765	
Overreliance on GAI Feedback (OGF)	OGF1	0.653	0.741	0.805
	OGF2	0.600	0.766	
	OGF3	0.628	0.753	
	OGF4	0.603	0.766	

This study conducted exploratory factor analysis (EFA) to examine the construct validity of the measurement items. The results are presented in Table 5. The Kaiser–Meyer–Olkin (KMO) value reached 0.940, indicating that the data were suitable for factor analysis. Bartlett’s test of sphericity was significant (*χ*^2^ = 2843.858, df = 55, *p* < 0.001), suggesting sufficient correlations among the measurement items. The EFA results identified three factors that corresponded to the proposed constructs: perceived cognitive load, cognitive offloading, and overreliance on GAI feedback. The three factors jointly explained 65.057% of the total variance. After rotation, the explained variance of the three factors was 23.422%, 22.152%, and 19.482%, respectively, indicating that the extracted factors provided adequate representation of the measurement structure. Regarding factor loadings, all items showed strong loadings on their intended factors, with values ranging from 0.563 to 0.781, exceeding the commonly accepted threshold of 0.50. Specifically, CO1–CO4 loaded primarily on Factor 2, PCL1–PCL3 loaded primarily on Factor 3, and OGF1–OGF4 loaded primarily on Factor 1. In addition, the communality values ranged from 0.556 to 0.736, indicating that each item shared sufficient variance with the extracted factors. These results demonstrate that the measurement items showed satisfactory construct validity and could effectively represent their corresponding variables.

**Table 5:** Validity testing.

Item	Loading			Communality (common variance)
	Factor 1	Factor 2	Factor 3	
CO1	0.247	0.776	0.179	0.695
CO2	0.335	0.698	0.208	0.644
CO3	0.199	0.739	0.282	0.665
CO4	0.330	0.563	0.361	0.556
PCL1	0.334	0.197	0.750	0.713
PCL2	0.187	0.301	0.781	0.736
PCL3	0.343	0.274	0.650	0.615
OGF1	0.737	0.245	0.270	0.676
OGF2	0.700	0.333	0.160	0.627
OGF3	0.711	0.228	0.283	0.638
OGF4	0.672	0.237	0.290	0.592
Eigenvalue (Unrotated)	5.623	0.804	0.729	–
Explained Variance (Unrotated)	51.118%	7.310%	6.629%	–
Cumulative Explained Variance (Unrotated)	51.118%	58.428%	65.057%	–
Eigenvalue (Rotated)	2.576	2.437	2.143	–
Explained Variance (Rotated)	23.422%	22.152%	19.482%	–
Cumulative Explained Variance (Rotated)	23.422%	45.574%	65.057%	–
KMO	0.940			–
Bartlett’s Test of Sphericity	2843.858			–
*df*	55			–
*p*-value	< 0.05			–

## 4 Results

To examine whether the relationships among variables differed across levels of metacognitive monitoring, this study classified participants into high and low metacognitive monitoring groups based on their responses to the metacognitive monitoring item. The classification criterion was determined according to the meaning of the response options on the five-point Likert scale. Scores of 1 and 2 indicate that students rarely engaged in metacognitive monitoring during learning, reflecting a relatively low level of monitoring ability. In contrast, scores of 3, 4, and 5 indicate that students showed at least a moderate level of metacognitive monitoring, suggesting that they were able to monitor and regulate their learning process to some extent. Therefore, students who selected 1 or 2 were classified into the low metacognitive monitoring group, whereas students who selected 3, 4, or 5 were classified into the high metacognitive monitoring group. Based on this classification criterion, 309 students were included in the high metacognitive monitoring group and 291 students were included in the low metacognitive monitoring group.

### 4.1 Descriptive statistical analysis

To understand how university students experience and respond to GAI-supported learning, this study first conducted descriptive statistical analysis of all measurement items. The analysis included the mean, standard deviation, skewness, kurtosis, and coefficient of variation (CV). These indicators provided an initial overview of students’ perceived cognitive demands, their use of GAI as external cognitive support, and their tendency to rely on GAI feedback during learning activities.

As shown in Table 6, university students demonstrated a relatively high level of cognitive offloading (CO) when using GAI for learning. The four items measuring cognitive offloading (CO1–CO4) showed mean values ranging from 3.575 to 3.832, with standard deviations ranging from 0.775 to 0.959. Among these items, CO2 showed the highest mean score (*M* = 3.832, SD = 0.775), indicating that students frequently used GAI to support their understanding of learning content. The results also show that students did not use GAI only for obtaining information but also relied on it to organize materials, understand concepts, and solve learning problems. Meanwhile, university students reported considerable perceived cognitive load (PCL) during learning activities involving GAI. The three items measuring perceived cognitive load (PCL1–PCL3) showed mean values ranging from 3.828 to 3.853, with standard deviations ranging from 0.812 to 0.840. Among them, PCL2 obtained the highest mean score (*M* = 3.853, SD = 0.812). These findings suggest that students often encountered substantial information-processing demands and mental effort when completing learning tasks. The results indicate that GAI use mainly occurred in situations where students faced complex learning requirements rather than simple information acquisition.

**Table 6:** Descriptive statistical analysis.

Item	S. D.	Sum	Std. error	Mean 95% CI (*LL*)	Mean 95% CI (*UL*)	IQR	Kurtosis	Skewness	Coefficient of variation (CV)
CO1	0.919	2145.000	0.039	3.498	3.652	1.000	0.369	−0.728	26.819%
CO2	0.601	2299.000	0.032	3.770	3.894	1.000	0.840	−0.735	20.232%
CO3	0.801	2178.000	0.037	3.558	3.702	1.000	0.420	−0.703	24.657%
CO4	0.757	2294.000	0.036	3.754	3.893	1.000	1.387	−0.978	22.752%
PCL1	0.706	2304.000	0.034	3.773	3.907	1.000	1.424	−0.946	21.875%
PCL2	0.660	2312.000	0.033	3.788	3.918	0.000	1.667	−0.964	21.077%
PCL3	0.680	2297.000	0.034	3.762	3.894	0.000	1.837	−1.070	21.540%
OGF1	0.714	2392.000	0.035	3.919	4.054	1.000	0.764	−0.824	21.200%
OGF2	0.692	2279.000	0.034	3.732	3.865	1.000	0.648	−0.708	21.903%
OGF3	0.721	2280.000	0.035	3.732	3.868	1.000	1.050	−0.853	22.348%
OGF4	0.822	2273.000	0.037	3.716	3.861	1.000	0.808	−0.785	23.926%

In addition, the results revealed that university students showed a certain degree of overreliance on GAI feedback (OGF) during learning. The four items measuring overreliance on GAI feedback (OGF1–OGF4) showed mean values ranging from 3.788 to 3.987, with standard deviations ranging from 0.832 to 0.906. OGF1 obtained the highest mean score (*M* = 3.987, SD = 0.845), suggesting that students tended to accept GAI feedback as an important reference when completing learning tasks. The similar scores across the four items indicate that students’ reliance on GAI feedback involved multiple aspects, including feedback acceptance, learning judgment, and task evaluation. The distribution characteristics further indicated that all measurement items showed acceptable statistical properties. The skewness values ranged from −1.070 to −0.703, and the kurtosis values ranged from 0.369 to 1.837, suggesting that the data did not show serious deviations from normal distribution. In addition, the coefficients of variation ranged from 20.232 to 26.819%, indicating sufficient differences among students’ responses. These results suggest that the measurement items could effectively capture individual differences in students’ experiences and behaviors related to GAI-supported learning. Overall, the descriptive results show that university students experienced considerable cognitive demands while learning with GAI and tended to use GAI as external cognitive support. At the same time, students also showed a tendency to depend on GAI feedback when making learning judgments.

### 4.2 Confirmatory factor analysis

To examine whether the measurement model was appropriate for students with different levels of metacognitive monitoring, confirmatory factor analyses (CFA) were conducted separately for the low and high metacognitive monitoring groups. Students who selected 1 or 2 on the metacognitive monitoring item were classified into the low metacognitive monitoring group (*n* = 291), whereas students who selected 3, 4, or 5 were classified into the high metacognitive monitoring group (*n* = 309). The CFA results were used to evaluate whether the measurement items adequately represented the three latent constructs, including perceived cognitive load (PCL), cognitive offloading (CO), and overreliance on GAI feedback (OGF). The results of the CFA analyses for both groups are presented in Tables 7, 8.

**Table 7:** CFA for group 1.

	UNSTD	S. E.	C. R.	*P*	STD	SMC	AVE	CR
PCL1 ← PCL	1.000				0.708	0.501	0.487	0.740
PCL2 ← PCL	0.936	0.091	10.346	***	0.691	0.477		
PCL3 ← PCL	0.976	0.094	10.388	***	0.694	0.482		
CO1 ← CO	1.000				0.710	0.504	0.481	0.787
CO2 ← CO	0.871	0.081	10.795	***	0.711	0.506		
CO3 ← CO	0.837	0.085	9.897	***	0.647	0.419		
CO4 ← CO	0.907	0.085	10.714	***	0.705	0.497		
OGF1 ← OGF	1.000				0.712	0.507	0.470	0.780
OGF2 ← OGF	1.006	0.098	10.212	***	0.660	0.436		
OGF3 ← OGF	1.019	0.097	10.487	***	0.679	0.461		
OGF4 ← OGF	1.099	0.103	10.643	***	0.690	0.476		

The symbol *** indicates statistical significance.

**Table 8:** CFA for group 2.

	UNSTD	S. E.	C. R.	*P*	STD	SMC	AVE	CR
PCL1 ← PCL	1.000				0.766	0.587	0.556	0.790
PCL2 ← PCL	0.954	0.076	12.613	***	0.751	0.564		
PCL3 ← PCL	0.903	0.075	12.097	***	0.720	0.518		
CO1 ← CO	1.000				0.702	0.493	0.517	0.811
CO2 ← CO	0.797	0.069	11.479	***	0.730	0.533		
CO3 ← CO	0.987	0.085	11.599	***	0.739	0.546		
CO4 ← CO	0.904	0.081	11.115	***	0.704	0.496		
OGF1 ← OGF	1.000				0.770	0.593	0.537	0.822
OGF2 ← OGF	0.870	0.069	12.655	***	0.729	0.531		
OGF3 ← OGF	0.919	0.072	12.764	***	0.735	0.540		
OGF4 ← OGF	0.926	0.077	11.966	***	0.693	0.480		

The symbol *** indicates statistical significance.

The model fit indices indicated that the three-factor measurement model showed an acceptable fit in both groups. For the low metacognitive monitoring group, the model demonstrated satisfactory fit (*χ*^2^ = 45.391, df = 41, *χ*^2^/df = 1.107, GFI = 0.972, AGFI = 0.955, CFI = 0.996, TLI = 0.995, RMR = 0.018, RMSEA = 0.019). Similarly, the high metacognitive monitoring group also showed acceptable model fit (*χ*^2^ = 80.200, df = 41, *χ*^2^/df = 1.956, GFI = 0.953, AGFI = 0.925, CFI = 0.975, TLI = 0.966, RMR = 0.027, RMSEA = 0.056). These results suggest that the proposed three-factor measurement model adequately represented students’ responses across different levels of metacognitive monitoring. The standardized factor loadings of all measurement items were significant (*p* < 0.001) in both groups. For the low metacognitive monitoring group, the standardized loadings ranged from 0.691 to 0.708 for PCL, from 0.647 to 0.711 for CO, and from 0.660 to 0.712 for OGF. For the high metacognitive monitoring group, the corresponding ranges were 0.720–0.766 for PCL, 0.702–0.739 for CO, and 0.693–0.770 for OGF. These findings indicate that the measurement items effectively represented their corresponding constructs in both groups.

The convergent validity and reliability of the constructs were further assessed using average variance extracted (AVE) and composite reliability (CR). For the low metacognitive monitoring group, the CR values of PCL, CO, and OGF were 0.740, 0.787, and 0.780, respectively, while the AVE values were 0.487, 0.481, and 0.470. Although the AVE values were slightly below 0.50, the constructs showed acceptable reliability, and all standardized factor loadings exceeded the recommended level. For the high metacognitive monitoring group, the CR values were 0.790, 0.811, and 0.822, and the AVE values were 0.556, 0.517, and 0.537, respectively. These results indicate satisfactory reliability and convergent validity of the measurement model across both groups.

### 4.3 Discriminant validity

Discriminant validity was assessed using Pearson correlations and the square root of average variance extracted (AVE) for both metacognitive monitoring groups. As shown in Tables 9, 10, the square root of AVE values for PCL, CO, and OGF were higher than the correlations among the constructs in both groups, indicating satisfactory discriminant validity. For the low metacognitive monitoring group, the square root of AVE values for perceived cognitive load (PCL), cognitive offloading (CO), and overreliance on GAI feedback (OGF) were 0.698, 0.694, and 0.686, respectively. The correlations among the three constructs ranged from 0.643 to 0.682, and all correlation coefficients were lower than the corresponding square root of AVE values. For the high metacognitive monitoring group, the square root of AVE values for PCL, CO, and OGF were 0.746, 0.719, and 0.733, respectively. The correlations among the constructs ranged from 0.674 to 0.695, which were also lower than the corresponding square root of AVE values. These results indicate that PCL, CO, and OGF represented distinct constructs across different levels of metacognitive monitoring.

**Table 9:** Discriminant validity-group 1.

	PCF	CO	OGF
PCF	0.698		
CO	0.643	0.694	
OGF	0.678	0.682	0.686

Blue numbers on the diagonal are square roots of AVE.

**Table 10:** Discriminant validity-group 2.

	PCL	CO	OGF
PCL	0.746		
CO	0.674	0.719	
OGF	0.683	0.695	0.733

Blue numbers on the diagonal are square roots of AVE.

### 4.4 Heterotrait-monotrait ratio

The heterotrait–monotrait ratio (HTMT) was further examined to assess the discriminant validity of the measurement model across different levels of metacognitive monitoring. As shown in Tables 11, 12, the HTMT values among perceived cognitive load (PCL), cognitive offloading (CO), and overreliance on GAI feedback (OGF) were below the recommended threshold of 0.90 in both groups, indicating satisfactory discriminant validity. For the low metacognitive monitoring group, the HTMT values between PCL and CO, PCL and OGF, and CO and OGF were 0.844, 0.893, and 0.870, respectively. All values were below the recommended threshold of 0.90, suggesting that the three constructs showed adequate discriminant validity. For the high metacognitive monitoring group, the corresponding HTMT values were 0.845, 0.849, and 0.856, respectively. These values also remained below 0.90, indicating that PCL, CO, and OGF represented distinct but related constructs among students with higher levels of metacognitive monitoring. Overall, the HTMT results supported the discriminant validity of the measurement model across both groups.

**Table 11:** HTMT (heterotrait–monotrait ratio) - group 1.

	PCF	CO	OGF
PCF	–		
CO	0.844	–	
OGF	0.893	0.870	–

**Table 12:** HTMT (heterotrait–monotrait ratio) - group 2.

	PCL	CO	OGF
PCL	–		
CO	0.845	–	
OGF	0.849	0.856	–

### 4.5 Structural model results

After validating the measurement model, this study further examined the structural relationships among perceived cognitive load (PCL), cognitive offloading (CO), and overreliance on GAI feedback (OGF) across different levels of metacognitive monitoring. Students who selected 1 or 2 on the metacognitive monitoring item were classified into the low metacognitive monitoring group (*n* = 291), whereas students who selected 3, 4, or 5 were classified into the high metacognitive monitoring group (*n* = 309). The structural equation models were estimated using AMOS 31.0, and the results for both groups are presented in Tables 13, 14.

**Table 13:** SEM results for group 1.

	UNSTD	S. E.	C. R.	*P*	STD
CO ← PCL	0.977	0.109	8.953	***	0.844
OGF ← CO	0.341	0.128	2.667	0.008	0.396
OGF ← PCL	0.557	0.155	3.588	***	0.560
OGF ← Gender	−0.006	0.048	−0.129	0.898	−0.006
OGF ← Age	−0.043	0.030	−1.429	0.153	−0.063
OGF ← UseFre	0.001	0.024	0.033	0.974	0.001
PCL3 ← PCL	1.000				0.693
PCL2 ← PCL	0.963	0.095	10.177	***	0.692
PCL1 ← PCL	1.028	0.099	10.378	***	0.709
CO1 ← CO	1.000				0.710
CO2 ← CO	0.870	0.081	10.795	***	0.711
CO3 ← CO	0.837	0.084	9.909	***	0.647
CO4 ← CO	0.905	0.085	10.708	***	0.704
OGF1 ← OGF	1.000				0.715
OGF2 ← OGF	0.996	0.098	10.191	***	0.656
OGF3 ← OGF	1.017	0.096	10.541	***	0.680
OGF4 ← OGF	1.094	0.103	10.667	***	0.689

The symbol *** indicates statistical significance.

**Table 14:** SEM results for group 2.

	UNSTD	S. E.	C. R.	*P*	STD
CO ← PCL	0.984	0.100	9.824	***	0.840
OGF ← CO	0.506	0.125	4.055	***	0.506
OGF ← PCL	0.490	0.145	3.382	***	0.419
OGF ← Gender	0.138	0.063	2.197	0.028	0.089
OGF ← Age	−0.072	0.039	−1.856	0.063	−0.075
OGF ← UseFre	0.081	0.043	1.908	0.056	0.077
PCL3 ← PCL	1.000				0.720
PCL2 ← PCL	1.051	0.089	11.840	***	0.747
PCL1 ← PCL	1.114	0.092	12.156	***	0.770
CO1 ← CO	1.000				0.705
CO2 ← CO	0.792	0.069	11.547	***	0.730
CO3 ← CO	0.982	0.084	11.683	***	0.739
CO4 ← CO	0.896	0.080	11.142	***	0.702
OGF1 ← OGF	1.000				0.777
OGF2 ← OGF	0.863	0.067	12.825	***	0.729
OGF3 ← OGF	0.910	0.070	12.918	***	0.734
OGF4 ← OGF	0.918	0.076	12.111	***	0.693

The symbol *** indicates statistical significance.

The structural model results showed a similar pattern across the two groups. First, perceived cognitive load significantly and positively predicted cognitive offloading in both the low metacognitive monitoring group (*β* = 0.844, *p* < 0.001) and the high metacognitive monitoring group (*β* = 0.840, *p* < 0.001). Therefore, H1 was supported in both groups. This finding indicates that when students experience greater cognitive demands during learning, they are more likely to transfer part of their cognitive activities to GAI. Although students with higher metacognitive monitoring may have stronger abilities to regulate their learning processes, complex academic tasks still require considerable cognitive resources. Therefore, students may use GAI as external cognitive support when they encounter difficulties in information processing, knowledge organization, or problem solving.

Second, cognitive offloading significantly and positively predicted overreliance on GAI feedback in both groups. Specifically, the effect was significant in the low metacognitive monitoring group (*β* = 0.396, *p* = 0.008) and the high metacognitive monitoring group (*β* = 0.506, *p* < 0.001). These results support H2. The findings suggest that students who increasingly transfer cognitive activities to GAI are more likely to rely on AI-generated feedback during learning. Although GAI can provide timely explanations and suggestions that facilitate learning, frequent dependence on external cognitive support may increase students’ tendency to accept AI feedback as an important basis for learning judgments.

Third, perceived cognitive load showed a significant positive effect on overreliance on GAI feedback in both groups. The standardized path coefficient was *β* = 0.560 (*p* < 0.001) for the low metacognitive monitoring group and *β* = 0.419 (*p* < 0.001) for the high metacognitive monitoring group. Thus, H3 was supported. This result indicates that higher cognitive demands may directly increase students’ reliance on GAI feedback. When students face complex learning tasks that require substantial mental effort, they may seek immediate assistance from GAI to obtain explanations, suggestions, and feedback, thereby increasing their dependence on AI-generated information.

The mediating role of cognitive offloading was further examined using bootstrap analysis with 2,000 samples. The results showed that the indirect effect of perceived cognitive load on overreliance on GAI feedback through cognitive offloading was significant in both groups. For the low metacognitive monitoring group, the indirect effect was 0.335, with a bias-corrected bootstrap confidence interval ranging from 0.069 to 0.611. For the high metacognitive monitoring group, the indirect effect was 0.426, with a confidence interval ranging from 0.149 to 0.648. Since both confidence intervals excluded zero, H4 was supported in both groups. These results indicate that cognitive offloading partially explains how perceived cognitive load contributes to students’ overreliance on GAI feedback.

In all, the structural model results indicate that the proposed relationships among perceived cognitive load, cognitive offloading, and overreliance on GAI feedback were consistent across students with different levels of metacognitive monitoring. Higher cognitive demands were associated with greater cognitive offloading, which further related to stronger reliance on GAI feedback. In addition, perceived cognitive load also showed a direct association with overreliance on GAI feedback. These findings support the proposed research model across both metacognitive monitoring groups.

## 5 Discussion

### 5.1 Comparison of results between the two groups

To examine whether metacognitive monitoring level influenced the relationships among perceived cognitive load (PCL), cognitive offloading (CO), and overreliance on GAI feedback (OGF), this study compared the structural paths between the low metacognitive monitoring group (Group 1) and the high metacognitive monitoring group (Group 2). The results showed that both groups supported the proposed structural model, but the strength of several paths differed across groups. These differences indicate that metacognitive monitoring may shape how students transform learning-related cognitive demands into GAI-supported behaviors.

The effect of perceived cognitive load on cognitive offloading showed a strong positive relationship in both groups. Specifically, the standardized path coefficient was *β* = 0.844 for Group 1 and *β* = 0.840 for Group 2. The similar effect sizes suggest that cognitive demands during learning represent an important reason for students to use GAI as external cognitive support. When undergraduate students encounter complex learning materials, intensive information processing requirements, or difficult academic tasks, they may use GAI to assist with information organization, knowledge understanding, and problem solving. This finding indicates that cognitive load plays a fundamental role in encouraging cognitive offloading, regardless of students’ metacognitive monitoring levels.

The relationship between cognitive offloading and overreliance on GAI feedback showed a different pattern between the two groups. The path coefficient was *β* = 0.396 in Group 1 and increased to *β* = 0.506 in Group 2. Compared with students with lower metacognitive monitoring, students with higher metacognitive monitoring showed a stronger association between their use of cognitive offloading strategies and reliance on GAI feedback. This result suggests that students with higher metacognitive monitoring may use GAI in a more deliberate manner when managing learning tasks. However, when they frequently transfer cognitive activities, such as information processing and evaluation, to GAI, their reliance on AI-generated feedback may also increase. Therefore, among students with higher metacognitive monitoring, cognitive offloading appears to play a more important role in explaining their reliance on GAI feedback.

The direct effect of perceived cognitive load on overreliance on GAI feedback also differed between the two groups. The path coefficient was *β* = 0.560 for Group 1 and *β* = 0.419 for Group 2. The stronger direct effect in Group 1 indicates that students with lower metacognitive monitoring may rely more directly on GAI feedback when they experience greater cognitive pressure during learning. When students face challenging tasks and perceive higher mental demands, they may seek immediate assistance from GAI to obtain explanations, solutions, or feedback. In comparison, students with higher metacognitive monitoring may regulate their learning process through more diverse strategies, which may reduce the direct influence of cognitive load on GAI feedback dependence.

The mediation analysis further revealed differences in the role of cognitive offloading between the two groups. The indirect effect of perceived cognitive load on overreliance on GAI feedback through cognitive offloading was 0.335 in Group 1 and 0.426 in Group 2. The larger indirect effect in Group 2 indicates that cognitive offloading plays a more prominent mediating role among students with higher metacognitive monitoring. For these students, cognitive demands may influence GAI feedback dependence mainly through their decision to use GAI as an external cognitive resource. In contrast, Group 1 showed a stronger direct relationship between perceived cognitive load and GAI feedback dependence, suggesting that cognitive pressure itself had a more immediate influence on their reliance on GAI feedback.

Overall, the comparison between the two groups demonstrates that metacognitive monitoring may influence the pathway through which students develop reliance on GAI feedback. Students with lower metacognitive monitoring showed a stronger direct association between cognitive load and GAI feedback dependence, whereas students with higher metacognitive monitoring showed a stronger relationship between cognitive offloading and GAI feedback dependence. These findings suggest that metacognitive monitoring does not simply determine whether students use GAI, but influences the process through which cognitive demands are managed and translated into GAI-supported learning behaviors.

### 5.2 Theoretical implications

This study provides important theoretical implications for understanding cognitive and behavioral adjustment processes in generative artificial intelligence (GAI) supported learning environments. Unlike previous studies that mainly focus on technology acceptance, usage intention, or learning outcomes, this study adopts a cognitive psychological perspective to examine how cognitive demands influence students’ cognitive offloading behaviors and subsequently contribute to overreliance on GAI feedback. The findings reveal the psychological mechanisms linking perceived cognitive load, cognitive offloading, and GAI feedback overreliance, providing a new perspective for explaining how individuals allocate cognitive resources when interacting with intelligent systems.

First, this study extends the application of cognitive offloading theory to the context of generative AI. Previous research on cognitive offloading has primarily examined how individuals use external tools to reduce memory demands, calculation efforts, and information-processing burdens. This study further demonstrates that, in GAI-supported learning environments, cognitive offloading represents not only a strategy for reducing immediate cognitive demands but also a process that may shape individuals’ interaction patterns with intelligent systems. When undergraduate students experience higher cognitive load, they are more likely to use GAI to undertake part of the information-processing and feedback-related tasks. Continuous reliance on such external cognitive support may further strengthen students’ dependence on GAI feedback. Therefore, this study expands cognitive offloading theory from traditional external support behaviors to ongoing cognitive regulation processes in human-AI interaction.

Second, this study enriches cognitive load theory by revealing its role in shaping technology-related dependence behaviors. Existing cognitive load research has mainly emphasized its influence on learning performance, task completion, and cognitive resource allocation. The present study extends this perspective by showing that cognitive load also affects how individuals choose and rely on external intelligent support. The findings indicate that perceived cognitive load can directly predict overreliance on GAI feedback and indirectly influence such reliance through cognitive offloading. This suggests that when individuals encounter cognitive pressure, they may not simply reduce their engagement with learning tasks; instead, they may redistribute cognitive demands through external intelligent systems, which may gradually influence their patterns of technology use.

Third, this study advances the understanding of the psychological mechanisms underlying overreliance on GAI feedback. Previous research on artificial intelligence use has largely focused on users’ acceptance, attitudes, and behavioral intentions, while less attention has been given to why individuals gradually depend on AI-generated information and suggestions. By incorporating cognitive offloading as a mediating mechanism, this study demonstrates that GAI feedback overreliance is not merely a consequence of technological characteristics but also reflects individuals’ cognitive responses to learning demands. The findings highlight that AI dependence has an important cognitive foundation, and its development should be examined through individual psychological processes.

Finally, this study extends the role of metacognitive monitoring theory in intelligent learning environments. The results show that students with different levels of metacognitive monitoring followed different pathways in developing GAI feedback overreliance. For students with lower metacognitive monitoring, perceived cognitive load showed a stronger direct association with GAI feedback overreliance, whereas for students with higher metacognitive monitoring, cognitive offloading played a more prominent mediating role. These findings suggest that metacognitive monitoring does not simply determine whether students use GAI, but influences how they regulate their cognitive relationship with intelligent tools. Thus, this study broadens the application of metacognitive theory by explaining individual differences in AI-supported cognitive regulation.

### 5.3 Practical insights

The findings of this study provide several practical implications for students, educators, and higher education institutions in the context of GAI-supported learning. By revealing how perceived cognitive load, cognitive offloading, and metacognitive monitoring jointly influence overreliance on GAI feedback, this study offers practical guidance for promoting more effective and balanced use of generative AI in academic settings.

First, the findings suggest that educators should pay attention to students’ cognitive demands during learning tasks when integrating GAI into educational practices. Since perceived cognitive load significantly increased both cognitive offloading and overreliance on GAI feedback, instructors should consider the difficulty and complexity of learning activities before introducing GAI-based assistance. Instead of encouraging students to use GAI simply as a shortcut for completing tasks, educators can design learning activities that require students to engage in analysis, reflection, and knowledge construction while using GAI as a supportive resource. Such an approach may help students benefit from AI assistance without excessively transferring cognitive responsibilities to the technology.

Second, this study highlights the importance of developing students’ appropriate cognitive offloading strategies. Cognitive offloading itself is not necessarily harmful, as it can help students manage complex learning demands and improve efficiency. However, the findings indicate that greater cognitive offloading is associated with stronger overreliance on GAI feedback. Therefore, educators should help students distinguish between productive use of GAI and excessive dependence on AI-generated information. Training programs can encourage students to use GAI for generating ideas, organizing information, and receiving supplementary explanations while maintaining independent judgment and critical evaluation of AI feedback.

Third, the findings emphasize the importance of strengthening students’ metacognitive monitoring abilities in GAI-supported learning environments. The results show that students with different levels of metacognitive monitoring followed different pathways in developing GAI feedback overreliance. Therefore, universities should provide learning guidance that improves students’ awareness of their own cognitive processes, including how to evaluate when GAI assistance is necessary, how to monitor their reliance on AI feedback, and how to adjust their learning strategies. Improving metacognitive monitoring may help students establish a more balanced relationship with GAI by encouraging active regulation rather than passive acceptance.

Finally, higher education institutions should develop clear guidance for responsible GAI use in academic contexts. Since GAI has become an increasingly accessible learning resource, universities should not focus only on restricting its use but should promote students’ ability to use AI effectively and critically. Educational policies and training programs should emphasize AI literacy, critical evaluation skills, and independent knowledge construction. These efforts can help students obtain the benefits of GAI while reducing the risk of excessive dependence on AI-generated feedback.

### 5.4 Limitations and future research

Although this study reveals the relationships among perceived cognitive load, cognitive offloading, metacognitive monitoring, and overreliance on GAI feedback, several aspects require further investigation.

First, the cross-sectional design of this study limits the ability to establish causal relationships among the variables. Although the structural equation model supports the hypothesized associations and mediation pathways, the findings should be interpreted as relationships among variables rather than causal effects. Students’ interactions with GAI may involve dynamic processes in which cognitive load, cognitive offloading strategies, metacognitive monitoring, and feedback reliance influence each other over time. Future studies could adopt longitudinal designs, experimental methods, or experience sampling approaches to further examine the developmental and causal mechanisms underlying students’ interactions with GAI.

Second, this study used a single-item measure to classify students’ metacognitive monitoring levels. Although this approach provides a concise indicator of students’ perceived metacognitive monitoring and facilitates comparisons between groups, it may not fully capture the complexity and multidimensional characteristics of metacognitive monitoring. Metacognitive monitoring involves various processes, including monitoring understanding, evaluating learning progress, and regulating cognitive strategies, which may not be sufficiently represented by a single item. Future research could employ validated multi-item scales or combine self-reported measures with behavioral indicators to obtain a more comprehensive assessment of students’ metacognitive monitoring abilities.

Third, the comparisons between the low- and high-metacognitive-monitoring groups in this study were based on differences in path coefficient patterns rather than formal statistical tests of between-group differences. Therefore, these results should be interpreted as variations in structural relationships across metacognitive monitoring levels rather than confirmed differences between groups. Future research could apply formal multi-group analysis approaches to further examine whether the strength of relationships among variables significantly differs across different levels of metacognitive monitoring.

Fourth, the present study mainly relied on self-reported questionnaires to measure students’ GAI use experiences, cognitive perceptions, and behavioral tendencies. Although self-reported measures are useful for capturing students’ subjective experiences, they may also be influenced by recall bias, social desirability, and individual interpretation differences, which may introduce potential common method bias. Future research could integrate multiple data sources, such as GAI interaction logs, learning behavior records, task performance data, and experimental measures, to provide a more objective understanding of students’ cognitive regulation processes during GAI use.

Finally, the participants in this study were recruited from a single university, which may limit the generalizability of the findings. Students from different universities, academic disciplines, and educational contexts may differ in their access to AI technologies, learning environments, and patterns of GAI engagement. Therefore, future studies should include samples from multiple institutions and diverse educational backgrounds to further validate the proposed model and examine its applicability across different learning contexts.

## 6 Conclusion

This study investigated the psychological mechanisms underlying undergraduate students’ overreliance on GAI feedback by integrating perceived cognitive load, cognitive offloading, and metacognitive monitoring into a unified research model. Based on survey data from university students, this study examined how learning-related cognitive demands influence students’ use of GAI and how cognitive offloading contributes to the formation of GAI feedback dependence. The findings provide a cognitive perspective for understanding students’ interaction with generative artificial intelligence in learning environments.

The results demonstrate that perceived cognitive load plays an important role in shaping students’ GAI-related behaviors. When students experience higher cognitive demands during learning, they are more likely to adopt cognitive offloading strategies by using GAI as external cognitive support. Cognitive offloading further contributes to overreliance on GAI feedback, indicating that students’ dependence on AI feedback is not only related to technological factors but also reflects their cognitive responses to learning pressure. In addition, perceived cognitive load directly influences overreliance on GAI feedback, suggesting that cognitive demands can affect students’ reliance on AI-generated information through both direct and indirect pathways.

Furthermore, this study reveals the role of metacognitive monitoring in shaping the relationship between cognitive demands and GAI feedback dependence. The comparison between groups with different levels of metacognitive monitoring shows that students follow different pathways when developing reliance on GAI feedback. Students with lower metacognitive monitoring show a stronger direct association between perceived cognitive load and GAI feedback overreliance, whereas students with higher metacognitive monitoring show a stronger role of cognitive offloading in explaining GAI feedback dependence. These findings suggest that metacognitive monitoring influences not only students’ learning regulation but also how they manage their cognitive relationship with intelligent tools.

Overall, this study extends the understanding of GAI-supported learning by demonstrating that overreliance on AI feedback emerges from the interaction between cognitive demands, external cognitive support, and self-regulatory processes. The findings highlight that effective GAI use requires more than access to intelligent tools; it also depends on students’ ability to regulate cognitive resources and evaluate AI-generated information appropriately. This study provides theoretical and practical insights into how university students can develop a more balanced and productive relationship with generative artificial intelligence in learning contexts.

## Data Availability

The raw data supporting the conclusions of this article will be made available by the authors, without undue reservation.
